# Rapid Brain Responses to Familiar vs. Unfamiliar Music – an EEG and Pupillometry study

**DOI:** 10.1038/s41598-019-51759-9

**Published:** 2019-10-30

**Authors:** Robert Jagiello, Ulrich Pomper, Makoto Yoneya, Sijia Zhao, Maria Chait

**Affiliations:** 1 Ear Institute, University College London, London, UK; 2 Present Address: Institute of Cognitive and Evolutionary Anthropology, University of Oxford, Oxford, UK; 3 Present Address: Faculty of Psychology, University of Vienna, Vienna, Austria; 4 NTT Communication Science Laboratories, NTT Corporation, Atsugi, 243-0198 Japan

**Keywords:** Perception, Auditory system

## Abstract

Human listeners exhibit marked sensitivity to familiar music, perhaps most readily revealed by popular “name that tune” games, in which listeners often succeed in recognizing a familiar song based on extremely brief presentation. In this work, we used electroencephalography (EEG) and pupillometry to reveal the temporal signatures of the brain processes that allow differentiation between a familiar, well liked, and unfamiliar piece of music. In contrast to previous work, which has quantified gradual changes in pupil diameter (the so-called “pupil dilation response”), here we focus on the occurrence of pupil dilation events. This approach is substantially more sensitive in the temporal domain and allowed us to tap early activity with the putative salience network. Participants (N = 10) passively listened to snippets (750 ms) of a familiar, personally relevant and, an acoustically matched, unfamiliar song, presented in random order. A group of control participants (N = 12), who were unfamiliar with all of the songs, was also tested. We reveal a rapid differentiation between snippets from familiar and unfamiliar songs: Pupil responses showed greater dilation rate to familiar music from 100–300 ms post-stimulus-onset, consistent with a faster activation of the autonomic salience network. Brain responses measured with EEG showed a later differentiation between familiar and unfamiliar music from 350 ms post onset. Remarkably, the cluster pattern identified in the EEG response is very similar to that commonly found in the classic old/new memory retrieval paradigms, suggesting that the recognition of brief, randomly presented, music snippets, draws on similar processes.

## Introduction

The human auditory system exhibits a marked sensitivity to familiar music^1–6^. The concept of music familiarity heavily relies on long term memory traces^7^, auditory mental imagery^8–10^ and is also linked to autobiographical memories, especially for emotionally relevant music^11^. Our prowess towards recognizing familiar musical tracks is anecdotally exemplified by “Name That Tune” games, in which listeners of a radio station are asked to name the title of a song on the basis of a very short excerpt. Even more fleeting recognition scenarios may occur when switching from one station to another while deciding which one to listen to—beloved songs often showing the ability to swiftly catch our attention, causing us to settle for a certain channel.

Here we seek to quantify, in a laboratory setting, one aspect of such recognition. Specifically, we aim to understand how quickly listeners’ brains can identify snippets from a familiar and personally relevant piece of music from among unfamiliar snippets, and pinpoint the neural signatures of this recognition. Beyond basic science, understanding the brain correlates of music recognition is useful for various music-based therapeutic interventions^12^. For instance, there is a growing interest in exploiting music to break through to dementia patients for whom memory for music appears well preserved despite an otherwise systemic failure of memory systems^13,14^. Pinpointing the neural signatures of the processes which support music identification may provide a clue to understanding the basis of the above phenomena, and how it may be objectively quantified.

Previous work using behavioral gating paradigms demonstrates that the latency with which listeners can identify a familiar piece of music (pop or instrumental) amongst unfamiliar excerpts ranges from 100 ms^5^ to 500 ms^3,15–17^. It is likely that such fast recognition is driven by our memory of the timbre and other spectral distinctivenesses of the familiar piece^15,18–20^.

According to bottom-up theories of recognition memory, an incoming stimulus is compared to stored information, and upon reaching a sufficient congruence is then classified as familiar^15,21^. A particular marker in the EEG literature that is tied to such recognition processes is the late positive potential (LPP)^22–24^: The correct identification of a familiar stimulus typically results in a sustained positivity ranging from 500 to 800 ms post-stimulation in left central-parietal regions, which is absent for unfamiliar stimuli^25^. This parietal “old versus new effect” has consistently been found across various domains, such as facial^26^ and voice recognition^27^ as well as paradigms that employed visually presented-^28^ and spoken-^29^ words as stimuli. In an fMRI study, Klostermann *et al*.^30^ used 2-second long excerpts of newly composed music and familiarized their participants with one half of the snippet sample while leaving the other half unknown. Subsequently, participants were exposed to randomized trials of old and new snippets and were asked to make confidence estimates regarding their familiarity. Correct identification of previously experienced music was linked to increased activity in the posterior parietal cortex (PPC). However, due to the typically low temporal resolution of fMRI, the precise time course of the recognition process remains unknown.

Pupillometry is also increasingly used as a measure of music recognition and, more generally, of the effect of music on arousal. This is part of a broader understanding that cognitive states associated with vigilance, surprise and processing effort can be gleaned from measuring task-evoked changes in pupil size^31–38^. Pupil dilation also reliably co-occurs with musical chills^39^–a physiological phenomenon evoked by exposure to emotionally relevant and familiar pieces of music^40^ and hypothesized to reflect autonomic arousal. Underlying these effects is the increasingly well understood link between non-luminance-mediated change in pupil size and the brain’s neuro-transmitter mediated salience and arousal network (specifically Acetylcholine and Norepinephrine)^41–46^.

In particular, abrupt changes in pupil size are commonly observed in response to salient^47,48^ or surprising^36,49,50^ events, including those in the auditory modality. Work in animal models has established a link between such phasic pupil responses and spiking activity within norepinephrine (NE, alternatively noradrenaline) generating cells in the brainstem nucleus locus coeruleus (LC). The LC projects widely across the brain and spinal cord^51,52^ and is hypothesized to play a key role in regulating arousal. Phasic pupil responses are therefore a good measure of the extent to which a stimulus is associated with increased arousal or attentional engagement^32,53,54^. Here we aim to understand whether and how music familiarity drives pupil responses.

Pupil dilations have received much attention in recognition paradigms, analogue to the previously elaborated designs in which participants are first exposed to a list of stimuli during a learning phase and subsequently asked to identify old items during the recognition stage^55^. When identifying old words, participants’ pupils tend to dilate more than when confronted with novel words, a phenomenon which is referred to as the *pupil old/new* effect^56–58^. Otero *et al*.^59^ replicated this finding using spoken words, thus extending this effect onto the auditory domain. The specific timing of these effects is not routinely reported. The bulk of previous work used analyses limited to measuring peak pupil diameter (e.g.^56,60–62^) or average pupil diameter change over the trial interval^57,59^. Weiss *et al*.^63^ played a mix of familiar and unfamiliar folk melodies to participants and demonstrated greater pupil dilations in response to the known as opposed to the novel stimuli. In this study, the effect emerged late, around 6 seconds after stimulus onset. However, this latency may be driven by the characteristics of their stimuli (excerpts ranged in length from 12 to 20 seconds), as well as the fact that melody recognition may take longer than timbre-based recognition.

Here we combine EEG and pupillometry to investigate the temporal dynamics of the physiological processes that underlie the differentiation of a familiar and personally relevant from an unfamiliar piece of music. In contrast to previous work, which has quantified changes in pupil diameter (the so-called “pupil dilation response”), we focus on pupil dilation events (see Methods). This approach is substantially more sensitive in the temporal domain and allowed us to tap early activity with the putative salience network.

Our experimental paradigm consisted of exposing passively listening participants to randomly presented short snippets from a familiar and unfamiliar song—a design that constitutes a simplified version of the above-mentioned real-world scenarios such as radio channel switching, though we note that we have reduced the memory demands considerably by using only one familiar and one unfamiliar song per subject. A control group, unfamiliar with all songs, was also used. We sought to pinpoint the latency at which brain and pupil dynamics dissociate randomly presented familiar from unfamiliar snippets and understand the relationship between brain and pupil measures of this process.

## Methods

### Participants

The participant pool encompassed two independent groups: A main group (N_main_ = 10; 5 females; Mage = 23.56; SD = 3.71) and a control group (N_control_ = 12; 9 females; Mage = 23.08; SD = 4.79). All reported no known history of hearing or neurological impairment. Two participants from the main group were excluded from the EEG analysis, as their respective pairs of familiar and unfamiliar songs elicited significant differences in EEG responses in the control group (see Results for details). This left 8 analyzed data sets (4 females; M_age_ = 23.62; SD = 3.96).

Experimental procedures were approved by the research ethics committee of University College London and were performed in accordance with the relevant guidelines and regulations. Written informed consent was obtained from each participant. Participants were paid for their participation.

### Stimuli and procedure

#### Preparatory stage

Members of the main group filled out a music questionnaire, requiring them to list five songs that they have frequently listened to, bear personal meaning and are evocative of positive affect. One song per subject was then selected and matched with a control song, which was unknown to the participant, yet highly similar in terms of various musical aspects, such as tempo, melody, harmony, vocals and instrumentation. Since we are not aware of an algorithm that matches songs systematically, this process largely relied on the authors’ personal judgments, as well as the use of websites that generate song suggestions (e.g. http://www.spotalike.com, http://www.youtube.com). We provided each participant with the name as well as a 1500 ms snippet of the matched song to confirm that they are indeed unfamiliar with it. Upon completion, this procedure resulted in ten dyads (one per participant), each containing one familiar and one unfamiliar song (see Table 1 for song details). All of the selected songs contained vocals, and pairs were matched according to the gender of the lead singer. Importantly, matching was also verified with the control group (see below).

**Table 1:** List of song dyads (“Familiar” and “Unfamiliar”) used in this study.

	Familiar Song	Control Song
1.	M83 - Midnight City https://www.youtube.com/watch?v=dX3k_QDnzHE	Postiljonen - Atlantis https://www.youtube.com/watch?v=MnkzAPUHrOg
2.	Chuck Berry - You never can tell https://www.youtube.com/watch?v=uuM2FTq5f1o	Jerry Lee Lewis - Great Balls of Fire https://www.youtube.com/watch?v=Jt0mg8Z09SY
3.	Barbra Streisand - The way we were https://www.youtube.com/watch?v=GNEcQS4tXgQ	Carole King - So far away https://www.youtube.com/watch?v=UofYl3dataU
4.	Kiss - Detroit Rock City https://www.youtube.com/watch?v=iZq3i94mSsQ	Black Sabbath - After Forever https://www.youtube.com/watch?v=WEsfskCX84s
5.	The Lumineers - Cleopatra https://www.youtube.com/watch?v=aN5s9N_pTUs	The Strumbellas - Shovels & Dirt https://www.youtube.com/watch?v=EJYtrvMxYVw
6.	Muse - Dead Inside https://www.youtube.com/watch?v=I5sJhSNUkwQ	The Vaccines - Dream Lover https://www.youtube.com/watch?v=X60NXmTbunk
7.	Nightwish - Élan https://www.youtube.com/watch?v=8cfGLKgT8S8	After Forever - Energize Me https://www.youtube.com/watch?v=ml8rNN2WAec
8.	Paolo Nutini - Candy https://www.youtube.com/watch?v=x3xYXGMRRYk	Josh Ritter - Good Man https://www.youtube.com/watch?v=C81SyunWMAQ
9.	Iron Maiden - The Number Of The Beast https://www.youtube.com/watch?v=WxnN05vOuSM	Riot - Riot https://www.youtube.com/watch?v=lvbFXo-M8ec
10	CHVRCHES - Recover https://www.youtube.com/watch?v=JyqemIbjcfg	Grimes - Oblivion https://www.youtube.com/watch?v=m5H-YlcMSbc

Song were matched for style and timbre quality as described in the methods section. The songs were selected based on input from the “main” group. The “control” group were unfamiliar with all 20 songs.

The main role of the control group is to rule out any acoustic differences between song-pairs that might contribute to differences in brain responses. Participants were selected for the control group based on non-familiarity with any of the ten dyads, therefore the distinction between familiar and unfamiliar conditions does not apply to them. To check for their eligibility before entering the study, they had the opportunity to inspect the song list as well as to listen to corresponding excerpts from the chorus (1500 ms). Due to the fact that it was exceedingly difficult to identify participants unfamiliar with all 20 songs, those recruited to the control group comprised of international students enrolled at UCL who were largely inexperienced with western popular music. While English was not their native language, all were proficient in understanding spoken English (as per UCL’s admission requirements). Note that 6 of the participants of the main group were also non-native English speakers.

#### Stimulus generation and testing paradigms

The beginning and end of each song, which typically constitute silent or only gradually rising or fading parts of the instrumentation, were removed. Both songs from each pair were then divided into snippets of 750 ms. Out of these, 100 snippets were randomly chosen for each song. These song snippets were then used in two paradigms: (1) a passive listening task and (2) an active categorization task. In the passive listening task, participants listened to the snippets from each dyad in random order whilst their brain activity was recorded with EEG and their pupil diameters with an infrared eye-tracking camera. Each particular snippet was presented once only. Participants were instructed to attentively listen to the music. We chose a passive stimulation paradigm because it mimics an everyday listening situation, in which participants can focus on the music without distraction. Each block contained 200 trials, 100 of each song presented in random order with an inter-stimulus interval (ISI) randomized between 1 and 1.5 seconds. This resulted in a total duration of roughly 6.7 minutes per block. Participants from the main group were presented with only one block (pertaining to the dyad that contained their familiar song and the matched non-familiar song). Participants from the control group listened to all 10 dyads (each in a separate block) for a total of 10 blocks which were presented in random order.

The active categorization task was also divided into 1 block per dyad. In each block, participants were presented with 20 trials, each containing a random pairing of snippets, separated by 750 ms. In half of the trials, both snippets were drawn from the same song. In the other half, one snippet was taken from the familiar and the other from the unfamiliar song. Participants were instructed to indicate whether the two snippets were from the same song or from different songs, by pressing the corresponding buttons on the keyboard. There was no time limit imposed on the response, and trials were separated by 750 ms following a button press. Same as for the passive listening task, participants from the main group performed only one block, associated with their pairing of “familiar/unfamiliar” songs. Participants from the control group completed 10 blocks in random order.

#### Procedure

Participants were seated, with their heads fixed on a chinrest, in a dimly lit and acoustically shielded testing room (IAC Acoustics, Hampshire, UK). They were distanced 61 cm away from the monitor and 54 cm away from two loudspeakers, arranged at an angle of 30° to the left and right of the subject. Participants were instructed to continuously fixate on a white cross on a grey background, presented at the center of a 24-inch monitor (BENQ XL2420T) with a resolution of 1366 × 768 pixels and a refresh rate of 60 Hz. They first engaged in the passive listening task followed by the active categorization task. For both tasks, participants were instructed to attentively listen to the snippets. EEG and pupil diameter were recorded during the first task, but not during the second, which was of purely behavioral nature.

### EEG acquisition, preprocessing and analysis

EEG recordings were obtained using a Biosemi Active Two head-cap 10/20 system with 128 scalp channels. Eye movements and blinks were monitored using 2 additional electrodes, placed on the outer canthus and infraorbital margin of the right eye. The data were recorded reference-free with a passband of 0.016–250 Hz and a sampling rate of 2048 Hz. After acquisition, pre-processing was done in Matlab (The MathWorks Inc., Natick, MA, USA), with EEGLAB^64^ (http://www.sccn.ucsd.edu/eeglab/) and FieldTrip software^65^ (http://www.ru.nl/fcdonders/fieldtrip/). The data were downsampled to 128 Hz, low pass filtered at 40 Hz and re-referenced to the average across all electrodes. The data were not high pass filtered, to preserve low-frequency activity^25^, which is relevant when analyzing sustained responses. The data were segmented into stimulus time-locked epochs ranging from −500 ms to 1500 ms. Epochs containing artefacts were removed on the basis of summary statistics (variance, range, maximum absolute value, z-score, maximum z-score, kurtosis) using the visual artefact rejection tool implemented in Fieldtrip. On average, 2.1 epochs per song pair in the main group and 4.9 epochs in the control group were removed. The larger number for the control group is probably a consequence of the longer session duration (10 blocks instead of a single block for the main group) and associated fatigue. Artefacts related to eye movements, blinks and heartbeat were identified and removed using independent component analysis. Subsequently, the data were averaged over epochs of the same condition and baseline-corrected (200 ms preceding stimulus onset). In the control group, averaging was done separately for each dyad, resulting in 20 time series (2 (familiar/unfamiliar) × 10 dyads).

A cluster-based permutation analysis, which takes spatial and temporal adjacency into account^65,66^ was used to investigate potential effects in the EEG responses. For both main and control participants, pairwise t-tests were calculated between ‘familiar’ and ‘unfamiliar’ snippets in all electrodes and over the entire epoch length. The significance threshold was chosen to control family-wise error-rate (FWER) at p = 0.05.

### Pupil measurement and analysis

Gaze position and pupil diameter were continuously recorded by an infrared eye-tracking camera (Eyelink 1000 Desktop Mount, SR Research Ltd.), positioned just below the monitor and focusing binocularly at a sampling rate of 1000 Hz. The standard five-point calibration procedure for the Eyelink system was conducted prior to each experimental block. Due to a technical fault that caused missing data, the initial seven control participants were excluded from the pupillometry analysis, leaving five valid participants (5 females, Mage = 23.21, SD = 4.37) in the control group. Note that this still resulted in ample data since each control subject completed 10 blocks, one for each dyad. No participant was excluded from the main group.

To make sure that pupil data are of high quality and reflect a consistent gaze position, samples in which gaze position exceeded 1° away from fixation were removed from the analysis. Across participants, 6% of the data were rejected in this way.

The standard approach for analyzing pupillary responses involves across trial averaging of pupil diameter as a function of time. This is usually associated with relatively slow dynamics^47,67–69^ which are not optimal for capturing potentially rapid effects within a fast-paced stimulus. Instead, the present analysis focused on examining pupil event rate. This analysis captures the incidence of pupil dilation events^42^, irrespective of their amplitude, and therefore provides a sensitive measure of subtle changes in pupil dynamics that may be evoked by the familiar vs. non-familiar stimuli. Pupil dilation events were extracted from the continuous data by identifying the instantaneous positive sign-change of the pupil diameter derivative (i.e. the time points where pupil diameter begins to positively increase). To compute the incidence rate of pupil dilation events, the extracted events were convolved with an impulse function (see also^42,70^), paralleling a similar technique for computing neural firing rates from neuronal spike trains^71^. For each condition, in each participant and trial, the event time series were summed and normalized by the number of trials and the sampling rate. Then, a causal smoothing kernel ω(τ) = α^2^ × τ × e^(−ατ)^ was applied with a decay parameter of α = 1/50 ms^70–72^. The resulting time series was then baseline corrected over the pre-onset interval. For each condition, the pupil dilation rate averaged across participants is reported here.

To identify time intervals in which the pupil dilation rate was significantly different between the two conditions, a nonparametric bootstrap-based statistical analysis was used^73^: For the main group, the difference time series between the conditions was computed for each participant, and these time series were subjected to bootstrap re-sampling (with replacement; 1000 iterations). At each time point, differences were deemed significant if the proportion of bootstrap iterations that fell above or below zero was more than 99% (i.e. p < 0.01). This analysis was conducted over the entire epoch duration. All significant intervals are reported.

Two control analyses were also conducted to verify the effects found in the main group. Firstly, permutation analysis on the data from the main group: in each iteration (1000 overall), 10 participants were selected with replacement. For each participant, all trials across conditions were randomly mixed and artificially assigned to the “familiar” or the “unfamiliar” condition (note that these labels are meaningless in this instance). This analysis yielded no significant difference between conditions. A second control analysis examined pupil dynamics in the control group. Data for each control participant consisted of 10 blocks (one per dyad), and these were considered as independent data sets for this analysis, resulting in 50 control datasets. On each iteration (1000 overall), 10 control datasets were selected with replacement from the pool of 50 and used to compute the mean difference between the two conditions. From here, the analysis was identical to the one described for the main group. This analysis also yielded no significant difference between conditions.

## Results

### EEG

**Figure 1:**
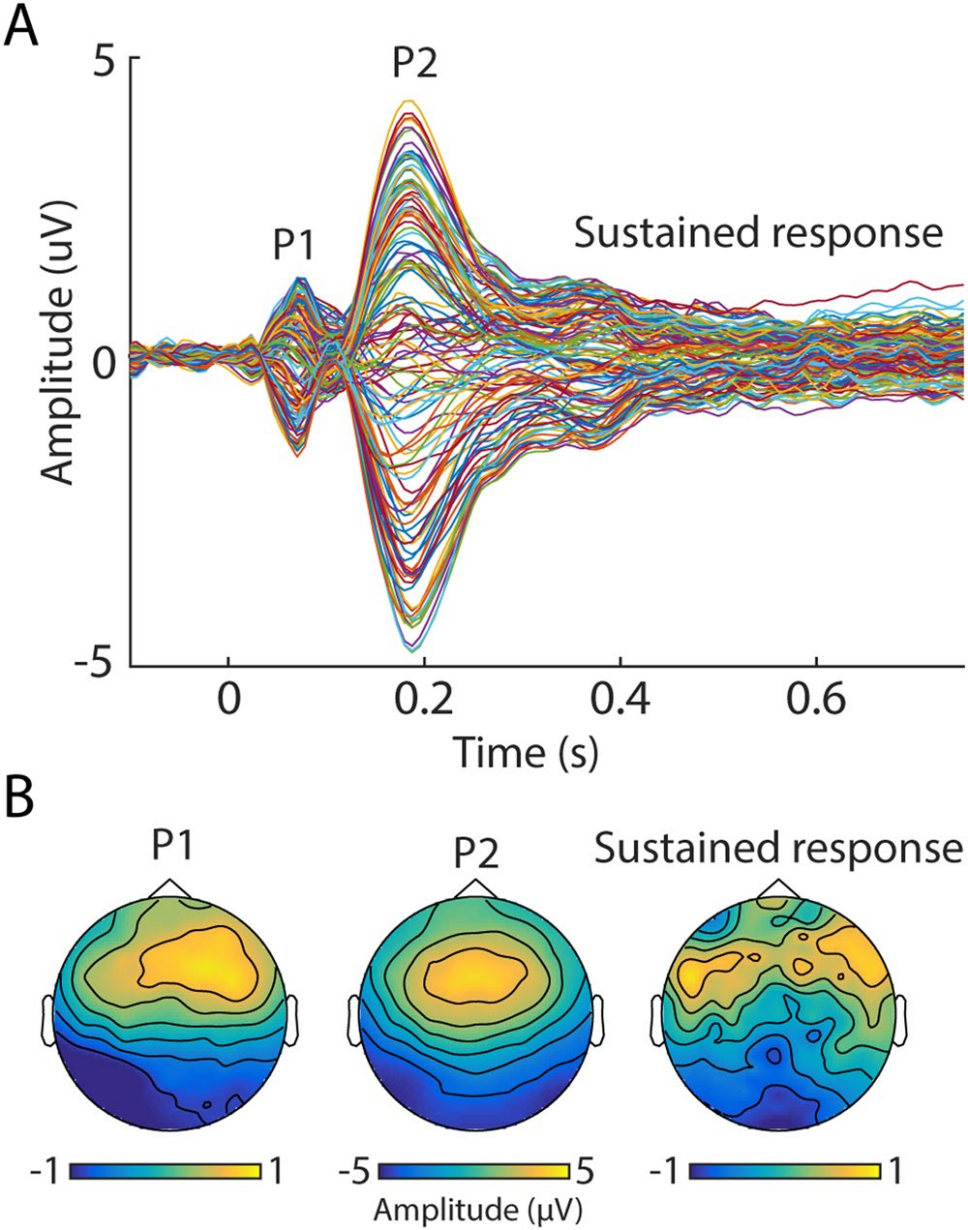
Grand-average event-related potential demonstrating the overall brain response to the music snippets. (**A**) Time-domain representation. Each line represents one EEG channel. The plotted data are averaged across the main and control groups, as well as familiar and unfamiliar songs. (**B**) Topographical representation of the P1 (71 ms), P2 (187 ms) peaks, as well as the sustained response (plotted is the average topography between 300–750 ms).

The overall EEG response to the sound snippets (collapsed across all participants and conditions) is shown in Fig. 1. The snippets evoked a characteristic onset response, followed by a sustained response. The onset response was dominated by P1 (at 71 ms) and P2 (at 187 ms) peaks, as is commonly observed for wide-band signals (e.g.^74^).

The main purpose of the control group was to verify that any significant differences which are potentially established for main participants were due to the manipulation of familiarity and not caused by any acoustic differences between the songs in each dyad. Because the control group participants were unfamiliar with the songs, we expected no differences in their brain responses to the songs in each dyad. However, the cluster-based permutation test revealed significant differences between conditions in dyad #2 (“familiar” more negative than “unfamiliar”) and #5 (“unfamiliar” more negative than “familiar”). This was taken as evidence that the songs were not matched properly, and the respective dyads (#2 and #5) were excluded from the subsequent main group analysis. Note that whilst this approach specifically targeted basic acoustic differences, it is possible, and indeed likely, that other, more abstract, differences between songs remain (e.g. differences in tonality or timbre). However, our use of different musical material for each dyad, spanning a wide range of genres (see Table 1), assures that any differences are not systematic across song material. This allows us to interpret the strongly consistent effects observed in the main group (see below) in terms of the symbolic difference between the “unfamiliar” and “familiar” songs, not linked to any physical stimulus parameters.

**Figure 2:**
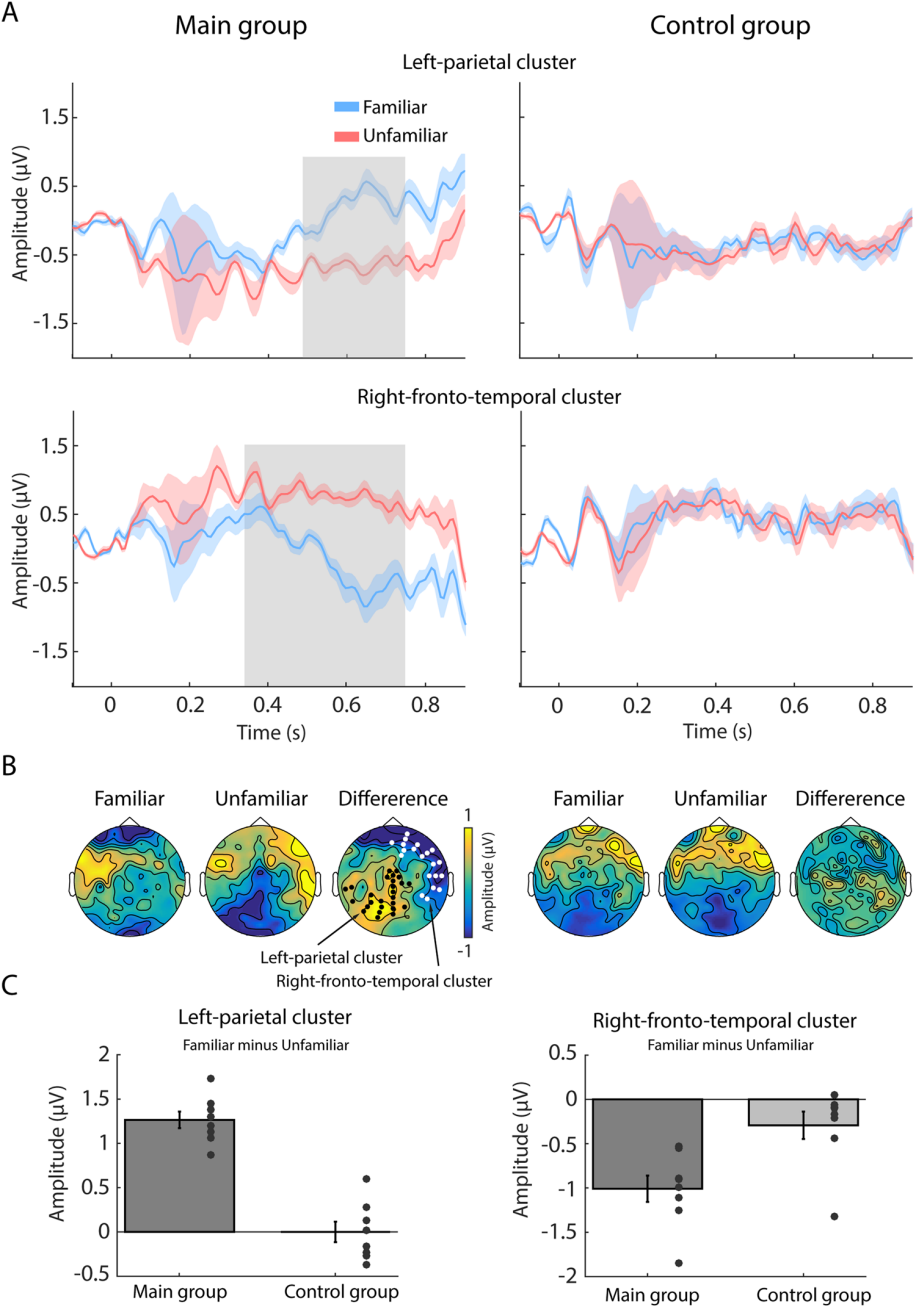
Event-related potential results – differences between “familiar” and “unfamiliar” snippets in the main, but not control, group. (**A**) Time-domain ERPs for the left-parietal cluster (top row) and right frontotemporal cluster (bottom row), separately for the main (left column) and control (right column) group. Solid lines represent mean data (averaged across channels and dyads) for familiar (blue) and unfamiliar (red) songs (note that this labelling only applies to the ‘main’ group; both songs were unfamiliar to the control listeners). Significant differences between conditions, as obtained via cluster-based permutation tests, are indicated by grey boxes. Note that the shaded areas reflect standard error of the mean for each condition, whilst the reported statistics are conducted as a repeated measures analysis. (**B**) Topographical maps of the “familiar” and “unfamiliar” ERP responses (computed from 350 to 750 ms) as well as their difference, separately for the main (left column) and control (right column) group. Black and white dots indicate electrodes belonging to the left-parietal and right frontotemporal cluster, respectively. (**C**) Mean ERP amplitude differences. The main group, but not the control group, showed significantly larger responses to unfamiliar song snippets, at both the left-parietal and the right frontotemporal clusters. Error bars represent standard error of the mean, dots represent mean response differences (across participants) to each song (8 “familiar” and 8 matched “unfamiliar” songs). In the main group, each dot reflects data from a single subject. In the control group, each dot reflects the average across the 12 members of the control group.

Comparing responses to “familiar” and “unfamiliar” snippets within the main group (Fig. 2), we identified two clusters of channels showing a significant difference between the two conditions (see Methods). A left-parietal cluster of 26 channels showing a significant difference between conditions from 540 to 750 ms (T_sum_ = −1238.71), and a right frontotemporal cluster of 20 channels, showing a significant difference between 350 to 750 ms (T_sum_ = 1127.41). These clusters are similar to those typically identified in old/new recognition memory studies^22,75–78^. Similar to what is observed here, the right frontotemporal cluster commonly exhibits earlier responses and is hypothesized to reflect familiarity, whereas the later responses in the left-parietal cortex are hypothesized to reflect retrieval^22^, consistent with dual-process theories of memory^79^.

To confirm that the observed differences are specific to the main group, we additionally performed a 2-factorial mixed ANOVA, with a within-subject factor of familiarity (familiar/unfamiliar) and a between-subjects factor of group (main/control). The average EEG amplitude across all channels and time points within the cluster served as the dependent variable. The interaction between the factors familiarity and group was significant for both the left-parietal cluster (F (1, 14) = 73.56, p < 0.001; partial η^2^ = 0.84) as well as the right frontotemporal cluster (F (1, 14) = 37.91, p < 0.001; partial η^2^ = 0.73). In both cases the intraction was driven by a significant difference between ‘familiar’ and ‘unfamiliar’ conditions in the main group, but a non significant difference among the control participants (left-parietal cluster: t_main_(7) = −13.7; p < 0.001; t_control_(7) = −0.03 p = 0.998; frontotemporal cluster: t_main_(7) = 8.14; p < 0.001; t_control_(7) = 1.3 p = 0.21). Note that the analysis presented so far was focused on dyads. Hence the control group data reflect data for each of 8 retained dyads, where responses to each song are averaged across the 12 members of the control group.

**Figure 3:**
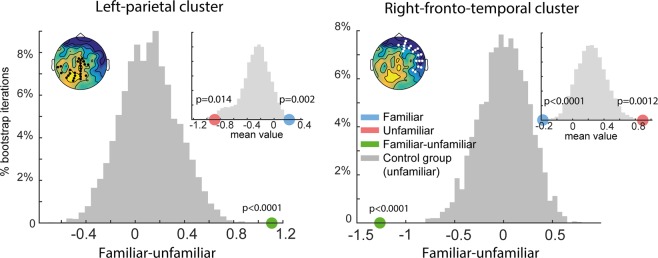
Bootstrap analysis comparing mean differences between “familiar” and “unfamiliar” responses from the main group to baseline difference distributions sampled from the control group (grey). As expected, difference distributions in the control group are centered around 0. Left upper insets show the respective electrode cluster overlaid on the ‘familiar – unfamiliar’ difference of the main group. In both clusters, responses observed in the main group (green dots) are well outside the baseline distributions (p < 0.001). Right upper inset figures show mean responses to “familiar” (blue dot) and “unfamiliar” (red dot) conditions from the main group, overlaid on distribution of responses from the control group (grey bars; collapsed across “familiar” and “unfamiliar”). Main group responses to both “familiar” and “unfamiliar” conditions are found on opposite sides of the control distributions, revealing a repulsive effect.

We also conducted a bootstrap resampling-based analysis to compare responses between the main group and matched subsets of the control group (Fig. 3). On each iteration (1000 overall) a single control subject was randomly assigned to each dyad (thus a subset of 8 control participants contributed 1 dyad each to the analysis). Data were then divided into “familiar” and “unfamiliar” conditions and averaged across participants (in the same way as for the main group; though the distinction between the two conditions is irrelevant for this group). Finally, the mean differences between “familiar” and “unfamiliar” conditions were computed over each of the ROI intervals. The grey histograms in Fig. 3 show the distribution of these values for each of the two ROIs (H0 distribution). The mean difference between conditions from the main group, indicated by the green dot, lies well beyond this distribution (p < 0.001), further confirming that the effect observed for the main group was different from that in the control group.

To understand how responses to “familiar” and “unfamiliar” music relate to those in the control group, we used a similar bootstrap routine, to obtain a distribution of mean control group responses in each ROI (collapsed across “familiar” and “unfamiliar” conditions) and compared to those in the main group. This analysis (Fig. 3; insets) demonstrates that responses to both “familiar” and “unfamiliar” snippets in the main group differed from those in the control group, such that, in both ROIs, “familiar” and “unfamiliar” responses lay on opposite edges of the control group distribution.

In terms of polarity and fieldmap distribution, main group responses to “unfamiliar” snippets were similar to the responses of the control group, though overall larger. It can be seen from the distributions plotted in Fig. 3 (insets) that the polarity of the “unfamiliar” response in the main group is consistent with the mean of the control group distribution. In contrast, the responses to familiar snippets in the main group lie on the opposite polarity and exhibit less deflection from 0. We will return to this point in the discussion.

### Pupil dilation

**Figure 4:**
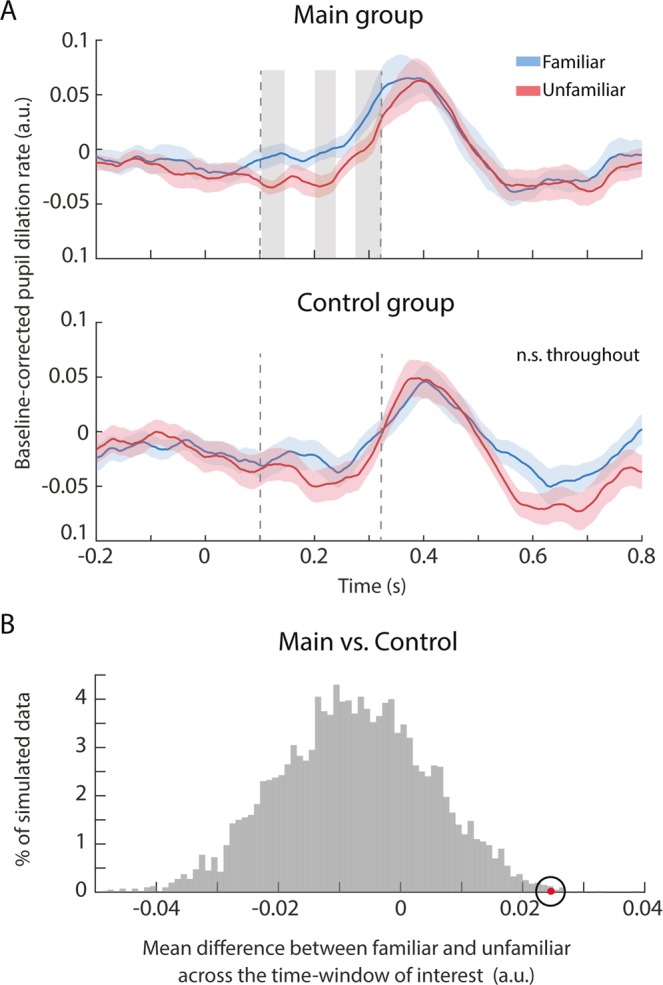
Pupil dilation rate to familiar and unfamiliar snippets. (**A**) Top: Main group. The solid curves plot the average pupil dilation rate across participants for “familiar” (blue) and “unfamiliar” (red) conditions. The shaded area represents one standard deviation from the bootstrap mean. The grey boxes indicate time intervals where the two conditions were significantly different (108–135, 206–232, and 282–319 ms). The dashed lines indicate the time interval used for the resampling statistic in (**B**). Bottom: Control group. The solid curves plot the average pupil dilation rate over 10 randomly selected control datasets. The shaded area represents one standard deviation of the bootstrap mean. No significant differences were observed throughout the entire epoch. (**B**) Results of the resampling analysis to compare the difference between “familiar” and “unfamiliar” conditions between the main and control groups (averages are computed over the time interval 108–319 ms, indicated via dashed lines in (**A**)). The grey histogram shows the distribution of differences between conditions for the control group (H0 distribution). The red dot indicates the observed difference in the main group.

Figure 4A (bottom) shows the pupil dilation rates for “familiar” and “unfamiliar” snippets in the control group. In response to the auditory stimuli, the pupil dilation rate increased shortly after the onset of a snippet, peaking at around 400 ms, before returning to baseline around 550 ms post-onset. No significant differences were observed between the two conditions throughout the entire epoch (see Methods), consistent with the fact that both were equally unfamiliar and hence equally salient to these participants.

In contrast, significant differences were observed in the main group. When compared with unfamiliar conditions, familiar snippets were associated with a higher pupil dilation rate between 108–319 ms post sound onset (Fig. 4A, top), i.e. during the rising slope of pupil dilation rate. This significant interval was absent in the shuffled data (see methods).

We also directly compared the difference between “familiar” and “unfamiliar” conditions between the two groups during the time interval (108–319 ms) identified as significant in the main group analysis. This was achieved by computing a distribution of differences between conditions based on the control group data (H0 distribution). On each iteration (1000 overall) 10 datasets were randomly drawn from the control pool and used to compute the difference between conditions during the above interval. The grey histogram in Fig. 4B shows the distribution of these values. The mean difference from the main group, indicated via the red dot, lies well beyond this distribution (p = 0.0034), confirming that the effect observed for the main group was different from that in the control group.

### Active categorization task

This task, conducted after the EEG and pupillometry session, aimed to verify whether participants were able to differentiate between familiar and unfamiliar snippets and whether participants in the main group (who were highly familiar with one song in a pair) performed better than controls.

Main participants correctly identified whether or not the two presented snippets were from the same song in 92% of trials, whereas controls did so in 79% of trials. An independent samples t-test revealed that main participants scored significantly higher than controls t(18) = 6.19, p < 0.00001. One-sample t-tests revealed that, in both groups, scores are at above-chance levels, t(9) = 13.61, p < 0.00001 for controls, and t(9) = 18.11, p < 0.000001 for the main group. Therefore, whilst there may have been enough information for control participants to consciously identify differences between snippets, this apparently did not affect the presently observed brain/pupil responses during passive listening.

## Discussion

We used EEG and pupillometry to identify brain responses which distinguish between a familiar, emotionally relevant and an unfamiliar piece of music. To tap rapid recognition processes, matched familiar and unfamiliar songs were divided into brief (750 ms) snippets which were presented in a mixed, random, order to passively listening participants. We demonstrate that despite the random presentation order, pupil and brain responses swiftly distinguished between snippets taken from familiar vs. unfamiliar songs, suggesting rapid underlying recognition. Specifically, we report two main observations: (1) pupil responses showed greater dilation rate to snippets taken from a familiar piece of music between ~100–300 ms post onset, and (2) brain activity measured with EEG showed differentiation between responses to familiar and unfamiliar music snippets from 350 ms post onset. The pattern of activation observed closely mirrored the ubiquitous old/new response patterns and thus suggest that similar mechanisms of recall and retrival were recruited.

The implications of these results for our understanding of the neural correlates of music recognition (see also^80^) are discussed below. But to start with, we outline several important limitations which the reader must keep in mind: Firstly, “familiarity” is a multifaceted concept. In the present study, songs were explicitly selected to evoke positive feelings and memories. Therefore, for the main group, the familiar and unfamiliar songs did not just differ in terms of recognizability but also in terms of emotional engagement and affect. Whilst we continue to refer to the songs as familiar and unfamiliar, the effects we observed may also be linked to these parameters. Future studies could further investigate potential interactions between these factors, by independently varying the familiarity and emotional valence of the presented music. Furthermore, the present experiment used only one familiar and one control song. This (relative to a case where multiple songs from each category are used) significantly reduced the demands on memory processes and might have allowed the brain to achieve discrimination based on maintenance of a template of the “familiar” song in some form of working memory.

Relatedly, it was inevitable that participants in the main group were aware of the aim of the study, and might have listened with an intent that is different from that in the control group. This limitation is difficult to overcome given the present research question, and the results must be interpreted in this light.

Furthermore, consideration must be given to the control group. These participants were required to be unfamiliar with all of the 20 songs used in the present study. Finding participants to satisfy this constraint is exceedingly difficult. We therefore resorted to recruiting international students (predominantly from Asia). As a consequence, their native language and musical experience differed from that of the main group (which comprised of participants from a European background). Note that, while all of the presented songs contained vocals, it is unlikely that semantic processing of the lyrics contributed to the observed effects. Since snippets were cut at random time-points within each song, many snippets did not contain a vocal passage at all. Those that did contain vocals were extracted at random points within words or syllables, eliminating any possible reliance on semantic processing.

Lastly, though we took great care in the song matching process, ultimately this was done by hand due to lack of availability of appropriate technology. Advancements in automatic processing of music may improve matching in the future. The control group was used to make sure that the familiar and unfamiliar songs were sufficiently acoustically matched. Because those participants were unfamiliar with either song in a dyad, we expected no differences in brain activity. Such was the case for 8 out of the 10 song pairs, i.e. two participants from the main group were discarded from further analysis. This highlights a further limitation of the present study which is associated with limited group size. Despite the various limitations, we note that the effects we observed (Figs 2 and 3) are large – suggesting the presence of a substantial, and robust, effect in the population.

Analysis of pupil dilation rates, demonstrated a characteristic rapid increase in rate evoked by snippet onset, peaking at roughly 400 ms. In the main group, we observed differences between responses to familiar and unfamiliar snippets during the rising slope of this response, such that the snippets taken from the familiar song evoked a larger increase in dilation rate, possibly reflective of recognition-linked surge in arousal.

The timing of this effect – between ~100–300 ms after onset - is broadly consistent with previous behaviorally derived estimates, which place minimum identification time for music at 100–250 ms^5,15^. Though they were listening passively, it is possible that the main group maintained a template of their familiar song in working memory and compared incoming snippets to this representation. For differentiation to happen so quickly, the relevant features were likely related to the timbre of the familiar song. It is known that humans possess a remarkable sensitivity to, and long term memory of, the timberal properties of complex sounds^19,20^ and these features are extracted early enough in the auditory processing hierarchy to be detectable within a short time of sound onset^19,20,81,82^.

Research in animal models has linked phasic pupil dilation events with increased firing in the LC^42^, hypothesized to reflect heightened arousal. Our approach of analyzing pupil dilation rates is particularly sensitive to capturing this activity. The present results can therefore be taken to indicate that the LC was differentially activated as early as ~100–300 ms after sound onset, possibly through projections from the inferior colliculus (where timbre cues may be processed^81,82^) to subcortical structures such as the hippocampus or amygdala, which are known to be linked to the LC^83,84^. Accumulating evidence^42,44^ demonstrates very rapid connectivity within this network, with IC-spike triggered pupil dilation events peaking at a latency of ~200 ms.

Our paradigm is conceptually similar to the old/new paradigms commonly used to investigate recognition memory^7,22,25^, but with important differences: Typical recognition memory studies test brain responses to recently memorized “neutral” stimuli which are usually static (e.g. words, faces, pictures). Here we tested brain responses associated with positive affect-based representations of temporally dynamic stimuli. These representations likely consist of current auditory templates of the familiar song, fed by emotionally charged long-term memory traces. It is possible that recognition would affect subsequent responses to the snippets as they unfold. Therefore, the observed EEG responses likely reflect both the process of recognition and the effect of familiarity on the processing of the ensuing portion of the sound. Additionally, unlike the standard old/new experiments, participants here listened passively and were not required to make a response, though, as mentioned above, it is possible that the main group were covertly making decisions about familiarity. For these reasons, comparison with previous results from the standard recognition memory paradigm may not be straightforward. It is noteworthy, however, that the cluster pattern identified in the EEG response is very similar to that commonly found in the classic old/new paradigms.

Specifically, in line with the ubiquitous findings in the memory literature, we blindly (i.e. using an unbiased whole scalp analysis) identified two clusters which distinguished familiar and unfamiliar responses: A right frontotemporal cluster, emerging from 350 ms after onset, and a left-parietal cluster emerging about 200 ms later - from 550 ms post onset. These responses are widely discussed in the memory literature as evidence for a two-stage memory process^85,86^ - an initial processing stage associated with familiarity, and later activation associated with recollection. In agreement with that literature, we observed more negative responses to “familiar” relative to “unfamiliar” items in the frontotemporal cluster, and the opposite (more positive for “familiar”) in the left-parietal cluster. This suggests that even short, randomly mixed, sound snippets can draw upon memory retrieval processes similar to those observed in active old/new judgment paradigms.

Important insight was obtained from comparing “familiar” and “unfamiliar” responses to responses from the control group. In that group, all stimuli were unfamiliar, and hence unlikely to have evoked old/new processing. Instead, responses presumably reflect “baseline” processing of unfamiliar snippets. We observed that main group activation to both familiar and unfamiliar snippets lay at opposite edges of the distribution of responses in the control participants. This “repulsive” effect suggests that both “familiar” and “unfamiliar” representations changed relative to the baseline afforded by the control group. We return to this point further below.

Overall, “unfamiliar” responses were more similar to the responses in the control group in that they shared polarity and field distribution, consistent with the fact that in both cases snippets were unfamiliar. In contrast, the “familiar” response was of opposite polarity to that exhibited by the control group, hinting at a qualitatively different process. Since scalp EEG is a reference-based measure, it is tricky to make direct claims about response magnitude. However, taking the distance from the 0 baseline as a measure of response energy, may suggest that more energy is expanded for processing the unfamiliar compared to the familiar items. In both the frontotemporal and parietal clusters, the magnitude of activation to the familiar snippets was smaller (closer to 0) than that to the unfamiliar snippets. One possible hypothesis, consistent with this pattern of results, is that, as discussed above, familiar snippets are recognized rapidly, mediated by fast-acting sub-cortical circuitry. This rapid dissociation between familiar and unfamiliar snippets may lead to later reduced cortical responses to the known stimulus and increased processing associated with the novel input e.g. as expected by predictive coding views of brain function^87–89^ whereby surprising, unknown stimuli require more processing than familiar, expected, signals.

The present study does not have sufficient data for reliable source analysis, however from the overall field maps (Fig. 2B) it appears that the identified clusters encompass the right superior temporal gyrus (rSTG), right inferior and middle frontal gyri (rIFG/rMFG) and left posterior parietal cortex (lPPC). Interestingly, a recent meta-analysis of fMRI work seeking to identify the neural correlates of music familiarity^80^, has identified broadly consistent brain regions.

The rSTG and rIFG/MFG have been implicated in processes related to recognition, notably in the context of voices^90,91^. Zäske *et al*.^92^ demonstrated that exposure to unfamiliar voices entailed increased activation in those areas. Similarly, old/new recognition paradigms have also demonstrated increased activation to unfamiliar (“new”) relative to familiar (“old”) words in these regions^93^. These increases in activation to unfamiliar items may be associated with explicit memory-driven novelty detection or else reflect a more general increase in activation related to attentional capture, or effort associated with processing of unfamiliar stimuli. Both the rIFG and rMFG have been implicated in a network that allocates processing resources to external stimuli of high salience/novelty^94–96^.

The left-paretial ROI is consistent with a large body of research which implicates left parietal regions (left posterior parietal cortex; lPPC) in episodic memory retrieval^7,22,76,97–101^. These areas are reciprocally connected to the para-hippocampal cortex and to the hippocampus, consistent with a role in the memory network, though the specific contribution of the PPC to episodic retrieval remain poorly understood.

Most reports, including EEG and BOLD-based investigations, found greater activation to familiar items in lPPC, which increases with the level of recollection of item details^7,30,100,102^. However, a recent human intracranial recording study^99^ revealed the presence of two types of memory selective neurons in the PPC: those that increased their firing rates for familiar stimuli, and those that preferred novel stimuli. The presence of memory sensitive cells that respond to unfamiliar items suggests that the coding of “newness” is associated with processing that is different from a mere absence of a familiarity signal. Our finding that main group responses to both familiar and unfamiliar items were altered relative to those in the control group, is in-line with these results and indicates that, rather than familiarity per se, the parietal cortex may reflect broader aspects of memory search. The overall strength of activation to familiar vs. unfamiliar items may be related to the specific task demands: paradigms in which memory strength is relatively weak (as is the case for most experiments that involve recently memorized items) may result in reliance on the “familiar” sensitive cells whilst tasks which probe robust memories and in which novel items are therefore more salient (such as may have been the case here) may prompt overall stronger responses to the novel items.

Together, the eye tracking and EEG data reveal early effects of familiarity in the pupil dynamics measure and later effects in the EEG brain responses. The lack of earlier effects in EEG may result from various factors, including that early brain activity may not have been measurable with the current setup. The audio snippets were cut from random parts of the song. Therefore, the temporal dynamics at onset differed between snippets, which may have resulted in phase misalignment of single-trial activations, reducing the aggregate evoked response. Failure to capture early brain responses may also arise due to non-optimal source orientation, or if the early effects do not arise in cortex. Thus, we suggest the observed latency of EEG responses to be an upper limit, with the actual earliest difference likely to arise much closer in time to the effect in pupil responses. In particular, as discussed above, the rapid pupillometry effects are likely to arise from sub-cortical recognition pathways and are therefore not measurable on the scalp. Future research combining sensitive pupil and brain imaging measurement is required to understand the underlying network.

In summary, the present results reveal that snippets from a familiar and unfamiliar song are differentiated rapidly in the brain. Even though only unique, randomly interleaved, snippets were presented, significant differences between responses were revealed from ~100–300 ms from sound onset. Initially, this was reflected by increased pupil dilation rate to the snippets from the familiar song. EEG responses differentiated from 350 ms after onset and recruited similar mechanisms to those previously identified in classic “old/new” memory paradigms. Both effects were remarkably stable across participants and song material (despite that different songs were used for each main subject). Due to the brief and random nature of snippet presentation, it is unlikely that these effects reflect recognition of melodic or semantic features but rather rely on a rapid match with a memory template of the familiar piece of music.

## Data Availability

The data supporting the results in this manuscript are available at [10.5522/04/9975983].
